# Stimulus arousal drives amygdalar responses to emotional expressions across sensory modalities

**DOI:** 10.1038/s41598-020-58839-1

**Published:** 2020-02-05

**Authors:** Huiyan Lin, Miriam Müller-Bardorff, Bettina Gathmann, Jaqueline Brieke, Martin Mothes-Lasch, Maximilian Bruchmann, Wolfgang H. R. Miltner, Thomas Straube

**Affiliations:** 1 Institute of Applied Psychology, School of Public Administration, Guangdong University of Finance, 510521 Guangzhou, China; 2 Institute of Medical Psychology and Systems Neuroscience, University of Muenster, 48149 Muenster, Germany; 3 Department of Clinical Psychology, Friedrich Schiller University of Jena, 07743 Jena, Germany

**Keywords:** Amygdala, Sensory processing

## Abstract

The factors that drive amygdalar responses to emotionally significant stimuli are still a matter of debate – particularly the proneness of the amygdala to respond to negatively-valenced stimuli has been discussed controversially. Furthermore, it is uncertain whether the amygdala responds in a modality-general fashion or whether modality-specific idiosyncrasies exist. Therefore, the present functional magnetic resonance imaging (fMRI) study systematically investigated amygdalar responding to stimulus valence and arousal of emotional expressions across visual and auditory modalities. During scanning, participants performed a gender judgment task while prosodic and facial emotional expressions were presented. The stimuli varied in stimulus valence and arousal by including neutral, happy and angry expressions of high and low emotional intensity. Results demonstrate amygdalar activation as a function of stimulus arousal and accordingly associated emotional intensity regardless of stimulus valence. Furthermore, arousal-driven amygdalar responding did not depend on the visual and auditory modalities of emotional expressions. Thus, the current results are consistent with the notion that the amygdala codes general stimulus relevance across visual and auditory modalities irrespective of valence. In addition, whole brain analyses revealed that effects in visual and auditory areas were driven mainly by high intense emotional facial and vocal stimuli, respectively, suggesting modality-specific representations of emotional expressions in auditory and visual cortices.

## Introduction

It has been suggested that the amygdala classifies sensory input according to its emotional and motivational relevance^1,2^ and modulates ongoing sensory processing leading to enhanced representations of emotionally relevant stimuli^3,4^. Social signals, such as emotional vocal and facial expressions, typically represent environmental aspects of high social and personal relevance (e.g., indicating other persons’ intentions or pointing towards relevant environmental changes) and high intense expressions are associated with higher arousal ratings as compared to low intense expressions^5^. It has been shown that the amygdala responds to both emotional vocal^6–9^ and facial expressions^10^. However, despite a large body of imaging studies on this issue, previous research does not provide an unequivocal answer regarding the factors that drive amygdalar responses to emotionally expressive voices and faces. Particularly, the specificity of amygdalar responding, that is, the proneness to respond to negative, threat-related emotional information has been a matter of debate^11–14^. Furthermore, it is uncertain whether emotional signals from different sensory domains are processed in an analogous fashion or whether modality-specific idiosyncrasies exist^15,16^.

Regarding the mentioned specificity of amygdalar activation to negative as compared to positive stimuli, findings have been mixed. Several studies employing emotional facial expressions suggest a heightened sensitivity for negative stimuli, threat-related stimuli in particular^17–23^. Unfortunately, most of these studies do not clarify, whether this ‘threat-sensitivity’ reflects effects of stimulus valence and/or stimulus arousal^19^. Several studies indicate that the amygdala is sensitive to positive and negative stimuli^24–27^ and might code general effects of motivational relevance and, therefore general arousal, irrespective of valence^11,12,14,28,29^. With regard to facial expressions, enhanced amygdalar activation has been observed for various types of facial expressions, including happy and surprised faces^23,30–32^. Previous research from our own lab provides evidence for amygdalar modulation as a function of stimulus arousal irrespective of stimulus valence by using positive and negative expressions of varying emotional intensity^5^. Here, intensity refers to the entirety of aspects, constitutive for the emotional experience as a whole^33,34^ and is highly correlated with emotional arousal^5^. Interestingly, some other studies also report modulation by expression intensity (and corresponding stimulus arousal), but report inverse intensity effects (that is, enhanced amygdalar activation to low intense/low arousing expressions^35^ (see discussion below).

With regard to affective voice processing, findings are also mixed. Using verbal and non-verbal vocalizations some studies show valence specific (e.g., responding to anger, fear, disgust but not happy vocalizations) amygdalar responses^36,37^, while others indicate valence-independent enhancements reflecting stimulus arousal^38,39^ or combined effects of stimulus valence and stimulus arousal^5^. In general, many studies only provide a dichotomous experimental manipulation (e.g., neutral versus negative expressions) and do therefore not provide information regarding separate contributions of stimulus valence and stimulus arousal^40–42^.

With respect to potential parallels between the processing of emotional vocalizations and facial expressions, it remains uncertain, whether the amygdala responds in a domain-general way across visual and auditory modalities. Recent reviews suggest that the amygdala is more important in affective face processing, as compared to the processing of emotional vocalizations^15,16^. On the other hand, a large number of imaging studies demonstrate enhanced amygdalar activation to emotional signals from the auditory compared to the visual domain^6,8,40,42,43^. In a similar vein, lesion studies also report impaired processing of emotional prosody in amygdala-lesioned patients^37,44–47^. Finally, there are some bimodal studies, which suggest analogous response patterns irrespective of the visual and auditory domains^36,48,49^. Aubé and colleagues (2015)^49^, for instance, demonstrate enhanced amygdalar activation in response to fear-related facial expressions, vocalizations and music plays, thus indicating parallels in the processing of emotional signals from different modalities^50^. Taken together, previous studies indicate that the amygdala responds to emotional signals from visual and auditory channels, although it is uncertain whether asymmetries in affective voice and face processing exist.

Several aspects might be relevant with regard to the heterogeneous findings of previous research. In particular, many of the above-mentioned studies neither assessed stimulus valence/arousal, nor controlled for comparable arousal levels across valence categories^22,36,49–51^. Positively-valenced facial expressions and voices may tend to be perceived as less arousing since they are frequently encountered in everyday life^28,52^. Importantly, several of the above-mentioned studies might have failed to create highly arousing positive signals – especially those which did not manipulate the intensity of emotional expressions^20,22^. These issues might reduce the arousal effect of positive expression on amygdalar responding, resulting in observing a valence-related effect or a combined effect of stimulus valence and arousal. In addition, only few studies used bimodal experimental designs including emotional signals from visual and auditory domains^36,49,50^, allowing for testing whether modality has an effect. Therefore, it remains uncertain, whether observed discrepancies reflect fundamental asymmetries in affective voice and face processing or methodological differences.

The present study aimed at systematically investigating the role of stimulus valence and stimulus arousal in the processing of emotional expressions from visual and auditory modalities. More precisely, we were interested in clarifying, whether amygdalar responding to affective voices and faces is driven by stimulus valence, stimulus arousal, or the interaction of both factors. In addition, we aimed to answer the question whether effects depend on the visual and auditory modalities of emotional expressions. In order to circumvent the abovementioned limitations of previous research, we employed stimuli, which provided different levels of emotional intensities for positive and negative expressions and therefore comprised varying levels of stimulus arousal and valence. Stimulus arousal was comparable between negative and positive expressions. In addition, rating data reflecting stimulus valence/arousal were used as parametric predictors, modeling brain activation based on stimulus specific mean arousal or valence ratings, in order to identify brain activation varying on these dimensions. Finally, we used a bimodal design in order to directly test potential domain-specific response patterns within the same experimental framework. Overall, we hypothesized that (1.) amygdalar responses reflect modulation of neuronal activation as a function of stimulus arousal with stronger activation for high arousing/high intense expressions, (2.) potential effects of stimulus arousal and expression intensity do not depend on stimulus valence and (3.) amygdalar responding as a function of stimulus arousal and stimulus intensity is analogous across visual and auditory modalities with no modality-specific idiosyncrasies.

## Methods

### Participants

Twenty healthy undergraduate and postgraduate students (19–28 years, *M* = 22.30, *SD* = 2.54; 10 females) were recruited from the University of Jena, Germany. Participants were right-handed as determined by the Edinburgh Handedness Inventory^53^. All participants had normal or corrected-to-normal vision and no participants had a history of neurological or psychiatric disease. The study was conducted in accordance with the guidelines of ethical standards in the Declaration of Helsinki and was approved by the Ethics Committee of the University of Jena. Written informed consent was obtained from all participants prior to participation.

### Stimuli

Facial and vocal expressions were selected from our newly developed stimulus sets, the Jena 3D Face Database (J3DFD) and the Person Perception Research Unit – EmoVoice (PPRU – EmoVoice), respectively. The J3DFD contains 32 Caucasian individuals showing angry, fearful, sad, disgusted, happy, and surprised expressions at three intensity levels plus neutral expressions^5,54^. The PPRU – EmoVoice database consists of twenty-four neutral bisyllabic nouns spoken in angry, fearful, sad, disgusted, happy, and surprised prosody at three intensity levels and a neutral prosody by five females and five males. Stimuli were recorded and digitized through an audio interface with a 44100 Hz sampling rate and 16 bit resolution and utterances were normalized in amplitude. These facial and vocal stimuli had been rated by independent samples of 44 and 50 participants, respectively, with respect to physiological arousal (ranging from 1 = very low to 9 = very high) and valence (ranging from 1 = very unpleasant to 9 = very pleasant). Emotional expressions were additionally rated with respect to the emotional expression intensity (ranging from 1 = very low to 7 = very high).

For the present study, we selected 50 facial and 50 vocal stimuli. Facial stimuli portrayed ten identities (5 females, 5 males) showing angry and happy expressions at high and low intensity levels plus neutral expressions. Vocal stimuli contained ten nouns spoken by 5 females and 5 males in angry, happy, and neutral prosodies, matched of stimulus duration per emotional category (mean: 658 ms, range: 415 ms − 917 ms, *F* (4, 45) = 0.84, *p* = 0.509, partial *η²* = 0.07). Mean ratings of emotional valence, arousal and intensity for facial and vocal stimuli are shown in Table 1. For arousal ratings, ANOVA analysis revealed no significant main effect of expression (*F* (4, 90) = 5.90, *p* = 0.057, partial *η²* = 0.86) or modality (*F* (1, 90) = 2.72, *p* = 0.175, partial *η²* = 0.41) but an interaction effect between expression and modality (*F* (4, 90) = 10.91, *p* < 0.001, partial *η²* = 0.34). Regarding valence ratings there was a significant main effect of expression (*F* (4, 90) = 8.83, *p* < 0.029, partial *η²* = 0.90) but not modality (*F* (1, 90) = 0.68, *p* = 0.457, partial *η²* = 0.15). Moreover, the interaction between expression and modality reached significance (*F* (4, 90) = 12.11, *p* < 0.001, partial *η²* = 0.35).

**Table 1:** Mean rating data on intensity (1 to 7), arousal (1 to 9) and valence (1 to 9) with respect to facial and vocal stimuli employed in the present study.

	Faces					Voices				
	Angry high	Angry low	Neutral	Happy low	Happy high	Angry high	Angry low	Neutral	Happy low	Happy high
Intensity	5.60 (0.60)	3.88 (0.76)		3.83 (0.60)	6.00 (0.43)	5.28 (0.66)	4.97 (0.75)		4.37 (0.74)	5.19 (0.52)
Arousal	5.93 (0.50)	4.48 (0.63)	2.24 (0.23)	4.11 (0.57)	5.85 (0.69)	4.39 (0.77)	4.22 (0.57)	2.64 (0.49)	3.86 (0.72)	4.19 (0.73)
Valence	2.29 (0.40)	3.21 (0.50)	5.30 (0.34)	6.76 (0.55)	6.58 (0.75)	3.22 (0.95)	3.41 (0.52)	4.96 (0.72)	5.04 (0.93)	5.61 (0.63)

*Note*: Values in parentheses represent standard deviations (*SD*).

### Procedure

**Figure 1:**
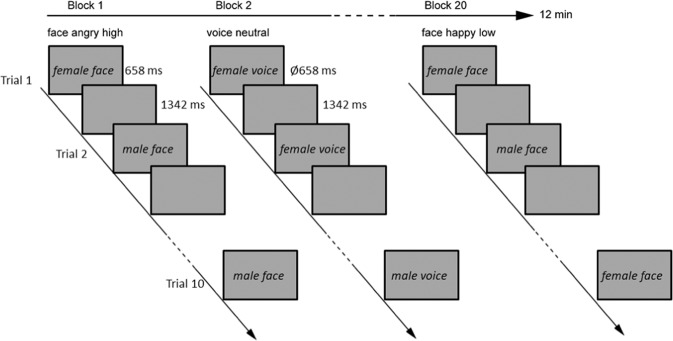
Each condition was presented in one block, consisting of ten trials. Visual stimuli were presented for 658 ms, while acoustic stimuli were in average presented for 658 ms with a stimulus onset asynchrony of 2000 ms. When presenting auditory stimuli, a blank screen was presented simultaneously. Each block was presented twice resulting in 20 blocks per run. Sequence of blocks were counterbalanced between runs and across participants. Participants were instructed to perform a gender judgment task in order to ensure that participants paid attention to the presented voices and faces.

Auditory stimuli were presented binaurally via headphones that were specifically adapted for the use in the fMRI environment (commander XG MRI audio system, Resonance Technology, Northridge, USA). When presenting auditory stimuli, a blank screen was presented simultaneously. Visual stimuli were shown via a back-projection screen onto an overhead mirror. Scanning was conducted in two runs (run duration 12 min). Overall, we had 10 conditions (2 modalities [faces vs. voices] × 5 expressions [angry high, angry low, neutral, happy low, happy high]). Each condition was presented in one block (see Fig. 1 for a schematic presentation of the procedure), consisting of ten trials (i.e., each facial/vocal identity [5 females, 5 males, see also the *Stimuli* section] was presented once in a block). The presentation sequence of each identity was randomized across blocks and participants. Each block was presented twice resulting in 20 blocks per run and overall, in 400 trials (5 expressions × 2 modalities × 10 identities × 2 repetitions × 2 runs). Between each block, there was an 18 second pause. Visual stimuli were presented for 658 ms, while acoustic stimuli were in average presented for 658 ms (see stimulus description) with a stimulus onset asynchrony of 2000 ms. Sequence of blocks were counterbalanced between runs and across participants. Participants were instructed to perform a gender judgment task in order to ensure that participants paid attention to the presented voices and faces. The instructions emphasized both speed and accuracy. Responses were given via button press of the index and the middle finger of the right hand, using a fiber optic response box (LUMItouch; Photon Control). Response assignments to index and middle finger were counterbalanced across participants. Only key pressing during stimulus presentation were considered as valid response. Stimulus presentation and recordings were accomplished by Presentation Software (Neurobehavioral Systems, Inc., Albany, California).

### Behavioral data recording and analysis

Accuracy and reaction times were analyzed with within-subject repeated measures analyses of variance (ANOVA) with the factors Modality (face and voice) and Expression (angry high, angry low, neutral, happy low, and happy high) using IBM SPSS 22 software (SPSS Inc., Chicago, Illinois). Greenhouse-Geisser and Bonferroni corrections were used, if appropriate. Results were regarded as statistically significant for *p* < 0.05.

### FMRI data acquisition and analysis

Scanning was performed in a 1.5-Tesla magnetic resonance scanner (Magnetom Vision Plus; Siemens Medical Systems). Following the acquisition of a T1-weighted anatomical scan, two runs of 245 volumes were obtained for each participant using T2*-weighted echo-planar images (TE = 50 ms, flip angle = 90°, matrix = 512 × 512, field of view = 200 mm, TR = 2973 ms). Each volume comprised 30 axial slices (thickness = 3 mm, gap = 1 mm, in-plane resolution = 3 × 3 mm). The slices were acquired parallel to the line between anterior and posterior commissure with a tilted orientation to reduce susceptibility artifacts in inferior parts of the anterior brain^55^. Before imaging, a shimming procedure was performed to improve field homogeneity. The first four volumes of each run were discarded from analysis to ensure steady-state tissue magnetization.

Preprocessing and analyses were performed using Brain Voyager QX (Brain Innovation, Maastricht, the Netherlands). The volumes were realigned to the first volume to minimize effects of head movements. Further preprocessing comprised spatial (8 mm full-width half-maximum isotropic Gaussian) and temporal (high-pass filter: three cycles per run, linear trend removal) filtering. The anatomical and functional images were co-registered and normalized to the Talairach space. The expected BOLD signal change for each predictor was modelled with a canonical double γ haemodynamic response function. The GLM was calculated with predictors of interest being the factors Modality (face and voice) and Expression (angry high, angry low, neutral, happy low, and happy high).

Valence and arousal effects were investigated using a parametric approach involving balanced contrast weights, which were derived from normative valence and arousal ratings reported in Table 1. Analysis was conducted for two main contrasts (valence and arousal) and their interaction with modality. For the first main contrast ‘arousal’, the arousal rating data for faces and voices were used as contrast weights, displaying a u-shaped function with higher values for high intense compared to low intense expression and neutral expressions being at the lowest point of the u-shape. Contrast weights were zero-centered. The second main contrast modeled valence effects by using normative valence ratings for faces and voices (see Table 1). This contrast modeled a linear function across expression predictors with positive values for positive valence. The two interaction contrasts of visual and auditory modalities with stimulus arousal or valence respectively were modeled using inverted contrast weights for voices. Interactions of arousal and valence were investigated with the mean-centered product of the mean-centered valance and arousal ratings. This parametric approach was chosen, since rating data reflecting stimulus valence/arousal were regarded as most accurate predictors for expected effects on amygdalar responses. Since contrast weights modelled brain activation separately for both modalities, we also controlled for potential differences across modality conditions.

Since the present study focuses on amygdalar response properties, data analysis was conducted as a region-of-interest (ROI) analysis for the amygdala. Additionally, to make the study more comprehensive, a whole-brain analysis was performed without a priori defined ROIs. The amygdala ROI was defined according to probabilistic cytoarchitectonic maps^56,57^ and contained the superficial group, the basolateral group, and the centromedial group as subregions^58^. Anatomical maps were created using the Anatomy Toolbox in Matlab (MATLAB 2014, The MathWorks, Inc., Natick, Massachusetts, USA) and transformed into Talairarch space using CBM2TAL^59,60^. Significant clusters were obtained through cluster-based permutation (CBP) with 1000 permutations. The non-parametric CBP framework was chosen, in order to gain precise false discovery rates with no need of assumptions regarding test-statistic distributions^61^. Voxel-level threshold was set to *p* < 0.005. For each permutation, individual beta maps representing activation patterns in a single experimental condition were randomly assigned without replacement to one of the tested experimental conditions. For example, to test the parametric arousal effect, the five beta maps corresponding to the five expressions were randomly assigned to these five conditions, separately for each subject. This approach is based on the assumption formulated by the null-hypothesis stating that the activation is equal across the five expression within a given subject. Cluster mass was assessed by summing all *t*-values in neighboring significant voxels, where voxels are defined as neighbors if they share a face (i.e. each voxel has six neighbors). Cluster masses larger than the 95% of the permutation distribution were considered as statistically significant.

## Results

### Behavioral results

#### Accuracy

Results revealed a significant main effect of Expression (*F* (4, 72) = 3.84, *p* = 0.007, partial *η²* = 0.18), which was further qualified by a significant two-way interaction between Modality and Expression (*F* (4, 72) = 5.37, *p* = 0.001, partial *η²* = 0.23). The main effect expression was significant for both voices (*F*(4, 76) = 5.45, *p* = 0.001, partial *η²* = 0.22) and faces (*F*(4, 76) = 3.35, *p* = 0.014, partial *η²* = 0.15). Bonferroni corrected post hoc *t*-tests revealed higher accuracy rates for angry high as compared to happy low expressions (*p* ≤ 0.001) for the visual domain and higher accuracy rates for happy low as compared to angry low and neutral expressions (*p’s* ≤ 0.004) for the auditory domain (see Table 2). No further contrast reached the Bonferroni corrected level of significance (all *p’*s > 0.05). There was no significant effect of modality (*p* = 0.299).

**Table 2:** Mean accuracy in percent and response times (RTs) in milliseconds for each experimental condition.

		Angry high	Angry low	Neutral	Happy low	Happy high
Accuracy	faces	76.38 (2.57)	73.13 (2.84)	74.38 (3.40)	69.00 (2.12)	74.75 (3.03)
	voices	71.10 (3.52)	63.70 (3.38)	63.75 (4.00)	73.60 (2.77)	70.75 (3.17)
RTs	faces	542.90 (7.25)	548.53 (10.95)	533.97 (9.36)	540.48 (8.55)	533.19 (8.53)
	voices	642.95 (6.30)	628.81 (7.55)	613.34 (8.25)	571.55 (10.61)	614.78 (6.21)

*Note*: Values in parentheses represent standard errors (*SE)*.

#### Response times

Results revealed main effects of Modality (*F* (1, 18) = 114.91, *p* < 0.001, partial *η²* = 0.87) and Expression (*F* (4, 72) = 10.80, *p* < 0.001, partial *η²* = 0.38, corrected). These main effects were further analyzed by a significant interaction between those two factors (*F* (4, 72) = 8.48, *p* < 0.001, partial *η²* = 0.32). The main effect expression was significant for voices (*F*(4, 76) = 12.92, *p* = 0.001, partial *η²* = 0.41), but not faces (*F*(4, 76) = 1.91, *p* = 0.118, partial *η²* = 0.09). Within the auditory domain, Bonferroni corrected post-hoc t-tests revealed shorter response times for happy low as compared to angry low, angry high, and neutral expressions, and for happy high as compared to angry high expressions (all *p’*s < 0.05).

### FMRI results

#### ROI analysis

**Figure 2:**
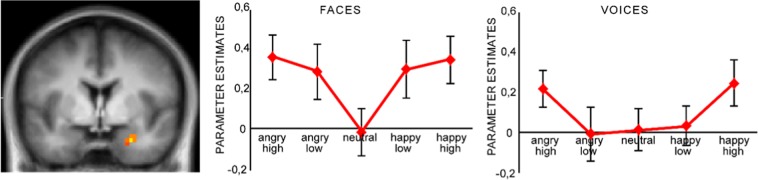
Enhanced activation in the right amygdala (x = 25, y = −4, z = −10) as a function of stimulus arousal for visual and auditory stimuli (CBP-corrected statistical map, initial voxel-level threshold *p* = 0.005). Bar plots show parameter estimates for visual (left side), and auditory (right side) stimuli. Parameter estimates refer to the mean cluster value, error bars indicate standard errors.

For the arousal contrast vector a significant activation cluster within the right amygdala was revealed, showing responses as a function of stimulus arousal (peak voxel coordinates: x = 25, y = −4, z = −10; *t*_*max*_ = 3.39, cluster mass = 18.68, *p* < 0.001, CBP corrected, cluster size = 6 voxels or 162 mm^3^, see Fig. 2). Importantly, there was no significant interaction between stimulus arousal and modality (*p* > 0.05). Furthermore, there were no significant clusters for the main contrast of valence as well as its interaction with stimulus modality (all *ps* > 0.05).

In order to additionally analyze whether or not there was an overall interaction between stimulus valence and stimulus arousal independent of modality, we used the mean-centered product of the mean-centered valence and arousal ratings as a contrast vector. There was no single voxel reaching the initial set voxel-level threshold. Finally, we also investigated potentially bimodal responses to valence^62^ by comparing all negative with all other stimuli und all positive with all other stimuli. There were no voxels that survived the voxel threshold.

#### Whole brain analysis

**Figure 3:**
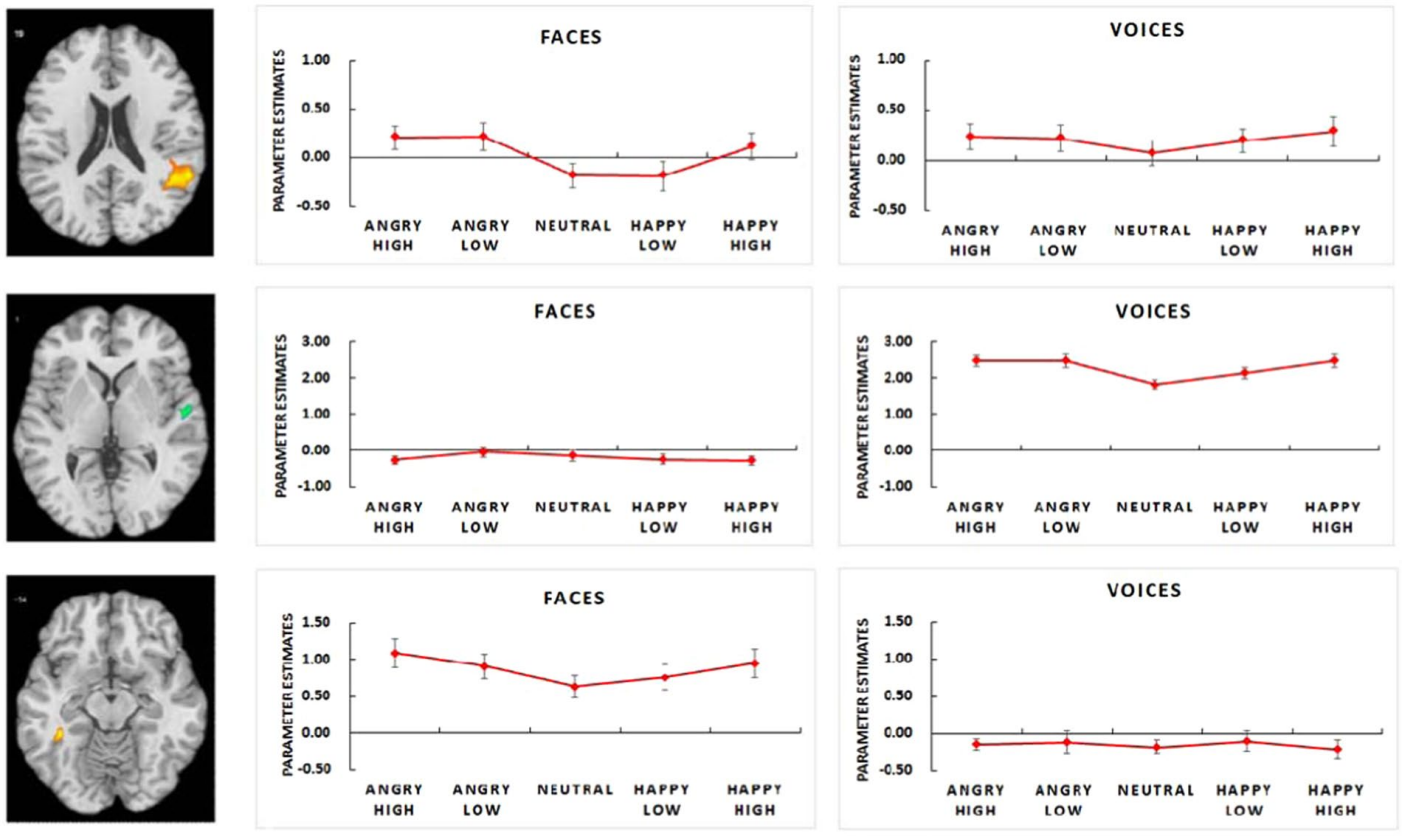
Significant activation cluster in posterior superior temporal sulcus (x = 48, y = −53, z = 18) as revealed by arousal contrast weights and significant activation clusters in medial superior temporal sulcus (x = 54, y = −16, z = 6) and fusiform gyrus (x = −39, y = −40, z = −8) as revealed by arousal × modality interaction contrast. Bar plots represent parameter estimates for arousal-driven effects in SMG, mSTS, and FG. Parameter estimates refer to peak voxels, error bars indicate standard errors.

There were several brain regions, which responded as a function of stimulus arousal, most importantly, mid superior temporal sulcus (STS, including the transversal gyrus), postcentral gyrus, posterior occipital cortex, insula, cingulate gyrus, and parts of the lateral frontal cortex (see Table 3 for a complete listing and Fig. 3 for main clusters).

**Table 3:** Significant activations modelled by the parametric arousal effect irrespective of visual and auditory modalities.

Region of activation	Hemisphere	x	y	z	Cluster mass	Cluster size (n voxels)	*t*(max)
Superior Temporal Gyrus	R	58	−50	18	1066.93	301	5.26
Superior Temporal Gyrus	R	48	−18	6	247.70	66	5.85
Middle Temporal Gyrus	R	53	−66	7	35.87	11	3.63
Inferior Frontal Gyrus	R	43	15	21	47.03	15	3.59
Inferior Temporal Gyrus	R	37	−60	−3	21.32	7	3.27
Middle Occipital Gyrus	R	24	−86	15	220.16	6	3.73
Superior Frontal Gyrus	R	22	27	44	24.63	69	3.31
Medial Frontal Gyrus	R	11	34	37	15.43	9	3.27
Cuneus	L	−1	−78	18	20.86	8	3.08
Cingulate Gyrus	L	−3	−9	38	31.61	5	3.65
Posterior Cingulate Gyrus	L	−6	−54	23	102.48	7	3.81
Cuneus	L	−4	−98	10	15.84	10	3.43
Middle Frontal Gyrus	L	−25	22	36	118.73	32	4.58
Insula	L	−31	−29	22	81.24	11	3.90
Middle Occipital Gyrus	L	−29	−76	10	21.93	5	3.56
Inferior Frontal Gyrus	L	−33	9	28	90.87	36	4.00
Middle Temporal Gyrus	L	−34	−65	19	15.25	5	3.13
Inferior Frontal Gyrus	L	−55	39	7	27.83	7	3.54
Postcentral Gyrus	L	−50	−25	22	19.13	25	3.38
Transverse Temporal Gyrus	L	−56	−22	10	31.62	7	3.43

*Note*. Significant activation clusters as identified by arousal contrast weights (*p* < 0.05, CBP corrected).

Clusters in the mid STS (x = 54, y = −16, z = 6) reflected modulation by vocal expression, while effects in fusiform gyrus (x = −39, y = −40, z = −8) reflected modulation by facial expression (see Fig. 3). Congruently, significant arousal × modality interactions were observed for these and several other brain regions, including supramarginal gyrus and anterior cingulate, indicating either preferred responses to voices or to faces (see Table 4 for a complete listing).

**Table 4:** Significant activations modelled by the parametric interaction of arousal and modality.

Region of activation	Hemisphere	x	y	z	Cluster mass	Cluster size (n voxels)	*t* (max)
**Positive clusters (faces** > **voices)**							
Postcentral Gyrus	R	42	−24	30	23.48	7	3.54
Supramarginal Gyrus	R	42	−41	31	87.84	24	5.21
Inferior Temporal Gyrus	R	37	−60	−3	114.45	32	4.33
Precentral Gyrus	R	31	−16	30	31.37	9	4.21
Middle Occipital Gyrus	R	30	−81	17	44.08	13	3.68
Middle Frontal Gyrus	R	32	23	38	188.89	51	4.92
Cuneus	R	17	−93	10	37.83	10	4.74
Anterior Cingulate Gyrus	R	22	43	4	59.00	17	4.29
Cingulate Gyrus	L	−3	−9	28	175.58	48	4.45
Medial Frontal Gyrus	L	−15	48	3	24.10	7	3.63
Middle Frontal Gyrus	L	−28	21	38	135.49	35	5.32
Caudate	L	−25	−25	30	50.80	14	4.57
Middle Frontal Gyrus	L	−31	−8	38	17.06	5	3.59
Caudate	L	−36	−25	−7	25.94	7	4.26
Fusiform Gyrus	L	−39	−40	−8	68.07	18	5.25
Inferior Occipital Gyrus	L	−40	−72	−3	16.84	5	3.54
**Negative clusters (faces** < **voices)**							
Superior Temporal Gyrus	R	54	−16	6	82.37	24	−3.84
Postcentral Gyrus	R	50	−32	49	17.70	5	−3.80
Middle Temporal Gyrus	R	48	−41	7	17.10	5	−3.78
Postcentral Gyrus	L	−50	−16	18	45.49	12	−5.07
Inferior Frontal Gyrus	L	−58	16	2	63.16	16	−4.87

*Note*. Significant activation clusters as identified by arousal × modality contrast weights (*p* < 0.05 CBP corrected). Negative *t*-values represent pattern with increased activity to faces compared to voices. The coordinates refer to the peak voxel in each cluster.

With regard to stimulus valence, significant clusters were mainly revealed in multi- and supramodal regions (e.g., insula, posterior STS, supramarginal gyrus, middle frontal gyrus), in visual areas (e.g., fusiform gyrus), and somatosensory areas (e.g., postcentral gyrus, see Table 5 for a complete listing of brain regions and Fig. 3 for main clusters). There were several significant valence × modality interactions, which reflected dominance for visually-driven valence effects (see Table 6 for a complete listing).

**Table 5:** Significant activations modelled by the parametric valence effect irrespective of visual and auditory modalities.

Region of activation	Hemisphere	x	y	z	Cluster mass	Cluster size (n voxels)	*t*(max)
Superior Temporal Gyrus	R	61	−31	12	143.99	39	−5.06
Inferior Occipital Gyrus	L	−40	−72	−3	97.64	28	−3.86
Supramarginal Gyrus	R	56	−50	18	231.97	60	−4.89
Superior Temporal Gyrus	R	51	−17	−1	171.07	45	−4.71
Middle Temporal Gyrus	R	56	−29	−1	23.96	7	−3.75
Insula	R	45	−39	16	78.85	20	−5.38
Inferior Frontal Gyrus	R	52	23	11	17.01	5	−3.65
Cingulate Gyrus	L	−7	27	42	62.20	17	−5.04
Medial Frontal Gyrus	L	−12	31	43	23.75	7	−3.90
Parahippocampal Gyrus	L	−24	−7	−29	17.66	5	−3.92
Middle Temporal Gyrus	L	−36	−59	19	188.99	55	−4.11
Middle Frontal Gyrus	L	−35	2	38	74.92	19	−5.76
Insula	L	−47	−36	24	127.53	34	−5.55
Insula	L	−42	−24	22	49.15	14	−3.95
Fusiform Gyrus	L	−41	−70	−11	16.76	5	−3.41
Middle Frontal Gyrus	L	−48	22	28	42.13	12	−3.93
Superior Temporal Gyrus	L	−45	−53	12	211.75	53	−6.57
Postcentral Gyrus	L	−55	−25	20	223.18	60	−4.95

*Note*. Significant activation clusters as identified by valence contrast weights (*p* < 0.05, CBP corrected). Negative t-values represent pattern with increased activity to angry compared to happy faces. The coordinates refer to the peak voxel in each cluster.

**Table 6:** Significant activations modelled by the parametric interaction of valence and modality.

Region of activation	Hemisphere	x	y	z	Clustermass	Cluster size (n voxels)	*t* (max)
**Negative clusters (faces** < **voices)**							
Postcentral Gyrus	L	−55	−25	20	23.96	7	−3.64
Superior Temporal Gyrus	R	50	−48	16	296.92	80	−4.83
Inferior Frontal Gyrus	R	44	2	26	170.60	46	−4.79
Inferior Temporal Gyrus	R	37	−63	−3	163.26	44	−4.97
Middle Frontal Gyrus	R	48	8	39	199.05	59	−3.80
Middle Frontal Gyrus	R	49	24	29	53.11	16	−3.47
Fusiform Gyrus	R	42	−51	−8	161.22	41	−5.42
Postcentral Gyrus	R	40	−26	27	39.39	11	−4.13
Middle Occipital Gyrus	R	39	−82	6	37.92	11	−3.78
Precentral Gyrus	R	36	4	36	41.04	12	−3.79
Insula	R	28	−30	19	20.58	6	−3.85
Middle Occipital Gyrus	R	24	−85	10	33.62	10	−3.55
Medial Frontal Gyrus	L	0	31	42	550.30	150	−5.08
Cuneus	R	12	−73	25	47.45	13	−4.28
Cingulate Gyrus	L	−7	−6	29	27.35	8	−3.80
Cuneus	L	−19	−86	22	59.57	17	−4.07
Superior Frontal Gyrus	L	−25	10	50	40.30	12	−3.84
Precentral Gyrus	L	−32	0	33	23.14	7	−3.42
Insula	L	−32	3	21	107.86	31	−4.00
Middle Occipital Gyrus	L	−37	−78	2	137.60	36	−5.49
Middle Frontal Gyrus	L	−37	29	43	16.81	5	−3.57
Middle Temporal Gyrus	L	−37	−62	19	17.49	5	−4.02
Middle Occipital Gyrus	L	−37	−64	2	42.78	12	−4.18
Middle Frontal Gyrus	L	−46	1	41	27.00	8	−3.58
Middle Frontal Gyrus	L	−49	25	28	91.99	26	−4.06
Inferior Frontal Gyrus	L	−48	16	19	16.30	5	−3.32
Superior Temporal Gyrus	L	−55	−50	10	26.83	8	−3.49

*Note*. Significant activation clusters as identified by valence × modality contrast weights (*p* < 0.05, CBP corrected). Negative t-values represent pattern with increased activity to faces compared to voices. The coordinates refer to the peak voxel in each cluster.

## Discussion

The present study investigated whether amygdalar responses to affective vocal and facial expression reflected modulation by stimulus valence and/or stimulus arousal. Furthermore, it was of interest whether or not potential modulation of the amygdala by valence and/or arousal would rely on analogous mechanisms for vocal and facial stimuli. We used voices and faces of varying emotional intensity across stimulus valence categories to examine this question. BOLD responses were modeled based on normative rating data on stimulus valence and arousal. Our results revealed amygdalar responses as a function of stimulus arousal and emotional intensity, crucially, irrespective of stimulus valence. In addition, arousal-driven effects for the amygdala were independent of the visual and auditory modalities of incoming emotional information, but reflected common response patterns across visual and auditory domains.

The proneness of the amygdala to respond to negative, threatening stimuli has been controversially debated^12,13^. Although enhanced amygdalar activation to negative, threat-related stimuli has been frequently observed^17,18,20,23,48^, there are few studies which provide convincing evidence in favor of valence-driven amygdalar responding (but see e.g., Kim *et al*.^19^). On the other hand, there is strong empirical support for the notion, that positive, negative, and ambiguous stimuli can elicit amygdalar responding, indicating that the amygdala shows general responsiveness to any salient emotional information^1,12,30^ and stimuli related to personal goals^2,25–27^. The present study adds to this observation indicating that amygdalar responses might code general stimulus relevance irrespective of stimulus valence and threat-relation.

There is also accumulating evidence that emotional intensity impacts amygdalar responding for several categories of emotional stimuli (e.g., scenes^34,63,64^ and odors^65,66^). In line with these studies, we find a significant positive relationship between amygdalar activation and stimulus arousal, and thus also a positive relationship between amygdalar activation and emotional intensity of facial expressions. Regarding facial expressions, several other studies found effects of emotional intensity on amygdalar responding^5,29,35^, which however varied. Interestingly, Gerber and colleagues^35^ observed inverse intensity effects, that is, enhanced amygdalar responding for weak, possibly ambiguous expressions. It is possible that the amygdala is sensitive to both stimulus intensity (signaling a need for prioritized processing) and stimulus ambiguity (signaling a need for gathering more sensory information), resulting in combined intensity and ambiguity effects^29^.

Even though there are many studies investigating whether amygdalar responses to vocal and facial expressions reflect modulation by stimulus valence or stimulus arousal, findings have been inconsistent so far^11–14^. Unfortunately, the majority of affective face and voice processing studies neither provide orthogonal manipulations of the two factors, nor include rating data on stimulus valence and arousal (but see e.g., Kim *et al*.^19^; Lin *et al*.^5^, for exceptions). In contrast to previous research, the present study provided highly arousing negative and positive expressions and systematically varied stimulus arousal and emotional intensity across emotional valence categories. Furthermore, statistical models were directly inferred from rating data on stimulus valence and arousal. Thus, our findings provide strong evidence that amygdalar responses to vocal and facial expressions reflect effects of emotional intensity and associated stimulus arousal and do not depend solely on stimulus valence.

Importantly, the present study also investigated whether amygdalar responses to stimulus arousal and expression intensity depend on the visual and auditory modalities of incoming information. The results of the present study provide evidence that the amygdala responds in an analogous fashion to social signals from visual and auditory modalities. These results are in line with earlier findings by Aubé and colleagues^49^, which suggest that the amygdala processes emotional information from different modalities in an analogous fashion. Our findings are also partly in line with the findings of Phillips and colleagues^36^, who found analogous amygdalar responses to fearful voices and faces (with respect to disgusted expressions, however, amygdalar enhancements were only observed for facial expressions). Interestingly, recent reviews proposed asymmetries in affective voice and face processing^15,16^. It is still uncertain, however, whether these asymmetries reflect minor relevance of subcortical structures in affective voice processing (as suggested by the authors) or methodological differences between the two research fields (e.g., less arousing vocal stimuli, smaller sample sizes, less sensitive statistical approaches in auditory studies). The present study experimentally manipulated stimulus modality as a within-subject factor and provided stimuli of comparable emotional properties across modalities. Controlling for methodological differences, we found parallel amygdalar response patterns for emotionally salient voices and faces. Thus, our results indicate that the amygdala responds in a domain-general fashion to emotional signals across visual and auditory domains with no modality-specific idiosyncrasies.

Besides the amygdala, our results provide evidence for domain-general, arousal-driven effects in several multimodal brain regions including the posterior STS, possibly indicating that these regions play an important role in the processing of stimulus arousal across visual and auditory modalities. A recent study by Lin and colleagues (2016)^5^ showed that stimulus arousal strongly impacts activation of the posterior STS in response to facial expressions. Several researchers proposed that the posterior STS is involved in the representation of facial information, particularly the representation of emotional expressions^67,68^, and demonstrated coupling with other face processing areas such as the fusiform gyrus^69,70^. Moreover, parts of the STS have been suggested to be the vocal analogue of the fusiform face processing area^9,71,72^, representing vocal features of varying complexity dependent on their emotional significance^8,9,71,73^. In addition, the posterior STS and supramarginal gyrus have been reported to be involved in the integration of audio-visual information and to respond to multiple types of social signals^74,75^. The results of the present study extend the findings of Lin and colleagues^5^ and indicate arousal-driven modulation of the posterior STS by facial and vocal expressions.

In addition, modality-specific arousal effects were observed in unimodal primary and secondary cortices, such as the lateral occipital cortex and the medial STS (mSTS), which showed enhanced activation in response to highly arousing faces and voices, respectively. In addition, modality-specific valence effects were also observed in some regions (see Table 5), which were primarily driven by visual stimulation, and reflected stronger activation to angry as compared to happy expressions. It is possible that advantages for the visual domain reflect a higher degree of specialization for representations of visual stimuli, in line with the dominance of visual representations in human perception. Mostly, modulation by stimulus valence did not reflect valence effects in isolation, but reflected mixed effects of stimulus valence and stimulus arousal, indicating limited empirical support for the valence model (see also Lindquist *et al*.^12^ for a recent meta-analysis on the plausibility of valence-driven brain responses).

There are several limitations of the present study. Since fMRI results were based on a 1.5 Tesla scanner, future work should investigate these issues with 3 or even 7 Tesla scanners and potential increased sensitivity for more nuanced effects^76–78^. We would like to mention that we do not suggest that the amygdala might not also code valence. However, the resolution of most fMRI studies makes it difficult to investigate this question in sufficient detail. Single unit studies provide also evidence for highly overlapping units with valence and arousal responses^79^. Future high resolution studies are needed to investigate the issue of potentially spatially distinct responses in small voxels due to valence, arousal, but also modality and other factors in more detail. Furthermore, the fact that the utilized auditory stimuli have no emotional meaning beyond prosody might be regarded as detrimental for the comparative validity of employed stimuli. Importantly, there are several studies demonstrating that it is rather prosody than meaning that causes an emotional reaction^80–83^. In addition, it should be noted that both stimulus categories provide affective and – to a large extend – non-affective information such as basic visual/auditory features related to gender, age, and identity. Considering these aspects, we regard the parallelism between the employed voices and faces as relatively far-reaching^15,16^. The present study used one specific negative emotion (i.e. anger) and a specific class of socially relevant stimuli. Thus, in order to ensure the generalizability of our findings to other types of negative expressions and emotional stimuli, the inclusion of a broader range of expressions^30^ and further emotional stimuli (e.g., biological emotional stimuli^84^) would be highly desirable. Finally, the present study found a valence-independent and modality-independent effect of arousal on amygdalar responding by using an implicit emotion task (e.g., a gender task). However, an explicit emotion task (e.g., an emotion discrimination task) is often used in studies on emotion processing. Furthermore, several studies have manipulated both explicit and implicit tasks to investigate the effect of task on the processing of emotional facial and vocal expressions^81,85,86^. Future studies might use both explicit and implicit tasks to investigate whether these tasks will show differential effects on arousal and valence dependent amygdala activations.

## Conclusion

Based on normative rating data on stimulus valence and arousal, the present fMRI study suggest enhanced amygdalar activation as a function of stimulus arousal, which does not depend on stimulus valence. Furthermore, present findings support the hypothesis of the amygdala as common neural substrate in affective voice and face processing, which evaluates emotional relevance irrespective of visual and auditory modalities. Finally, whole brain data provided evidence for modality-specific representations of emotional expressions in auditory and visual cortices, which again, mainly reflected the impact of emotional intensity and associated stimulus arousal. Future high resolution studies, however, should further investigate potential overlapping and distinct activations in the amygdala depending on arousal, valence, stimulus modality and specific task contexts.
